# Whey Proteins Are More Efficient than Casein in the Recovery of Muscle Functional Properties following a Casting Induced Muscle Atrophy

**DOI:** 10.1371/journal.pone.0075408

**Published:** 2013-09-19

**Authors:** Vincent Martin, Sébastien Ratel, Julien Siracusa, Pascale Le Ruyet, Isabelle Savary-Auzeloux, Lydie Combaret, Christelle Guillet, Dominique Dardevet

**Affiliations:** 1 Clermont Université, Université Blaise Pascal, EA 3533, Laboratoire des Adaptations Métaboliques à l'Exercice en Conditions Physiologiques et Pathologiques, CRNH Auvergne, Clermont-Ferrand, France; 2 Lactalis Recherche et Développement, Retiers, France; 3 INRA, Unité de Nutrition Humaine (UNH, UMR 1019), CRNH Auvergne, Clermont-Ferrand, France; 4 Clermont Université, Université d’Auvergne, Clermont-Ferrand, France; University of Valencia, Spain

## Abstract

The purpose of this study was to investigate the effect of whey supplementation, as compared to the standard casein diet, on the recovery of muscle functional properties after a casting-induced immobilization period. After an initial (I0) evaluation of the contractile properties of the plantarflexors (isometric torque-frequency relationship, concentric power-velocity relationship and a fatigability test), the ankle of 20 male adult rats was immobilized by casting for 8 days. During this period, rats were fed a standard diet with 13% of casein (CAS). After cast removal, rats received either the same diet or a diet with 13% of whey proteins (WHEY). A control group (n = 10), non-immobilized but pair-fed to the two other experimental groups, was also studied and fed with the CAS diet. During the recovery period, contractile properties were evaluated 7 (R7), 21 (R21) and 42 days (R42) after cast removal. The immobilization procedure induced a homogeneous depression of average isometric force at R7 (CAS: − 19.0±8.2%; WHEY: − 21.7±8.4%; P<0.001) and concentric power (CAS: − 26.8±16.4%, P<0.001; WHEY: − 13.5±21.8%, P<0.05) as compared to I0. Conversely, no significant alteration of fatigability was observed. At R21, isometric force had fully recovered in WHEY, especially for frequencies above 50 Hz, whereas it was still significantly depressed in CAS, where complete recovery occurred only at R42. Similarly, recovery of concentric power was faster at R21 in the 500−700°/s range in the WHEY group. These results suggest that recovery kinetics varied between diets, the diet with the whey proteins promoting a faster recovery of isometric force and concentric power output as compared to the casein diet. These effects were more specifically observed at force level and movement velocities that are relevant for functional abilities, and thus natural locomotion.

## Introduction

Chronic periods of reduced physical activity occur throughout life, following injuries, trauma, prolonged immobilization (casting or extended bed rest), or as a natural progression of ageing. The primary effect of muscle disuse in such situations is the progressive loss of skeletal muscle mass, even in the absence of disease [1]–[3], which is linked to an alteration of the mobility and movements. During rehabilitation, it is then of great importance to restore as fast as possible, not only skeletal muscle mass but also muscle functional properties, *i.e.* strength, power and fatigability.

Muscle protein loss results from an imbalance between protein synthesis and breakdown rates. We and others have demonstrated in rats and humans that muscle protein synthesis was depressed during immobilization [4]–[8] and that muscle proteolysis was highly activated during disuse atrophy [9]–[14] hence generating a decrease in muscle protein content and a loss of muscle mass. To allow muscle recovery, not only a normalization of muscle protein synthesis/proteolysis ratio is necessary but the generation of a positive nitrogen balance is also needed. It is now well established that skeletal muscle proteolysis normalizes very rapidly during the first days of reloading [12]–[14]. Therefore, the stimulation of protein synthesis during the recovery period is the major determinant of muscle mass recovery.

Resistance exercise is highly efficient to prevent muscle protein loss during immobilization or to accelerate muscle recovery [15]. However, exercise is not always relevant in specific clinical situations such as invalidating diseases, muscle traumatism, joint pain or frail elderly for example. Therefore, nutrition represents an alternative and/or complementary strategy to accelerate muscle mass recovery following bed rest or immobilization. Because of its signal properties toward muscle protein synthesis [16]–[19], increased availability of leucine or branched chain amino acids (BCAA =  leucine+valine+isoleucine) has been tested. However, BCAA or leucine supplementation led to conflicting results with either no or little impact [20] or a positive effect [21], [22] on protein synthesis and muscle function and strength. Very recently, we also reported no effect of such supplementation on muscle mass recovery following cast immobilization in old animals [7], despite a positive effect on protein anabolism following food intake. We postulated [23] that leucine, if supplemented in a free form, was absorbed rapidly and induced its anabolic ‘leucine signal’ for protein synthesis before there was a sufficient availability of amino acids coming from dietary protein digestion. However, when leucine-rich proteins (whey proteins) were used instead of free leucine, an improvement of muscle mass recovery was observed, probably because leucine and other amino acids were elevating synchronically and generated a more sustained protein synthesis stimulation during the post prandial state [7], [23]. According to this unique study, whey proteins seem to be a suitable nutritional strategy to accelerate muscle mass recovery following immobilization or physical inactivity episodes.

So far, the great majority of nutritional interventions aimed at promoting muscle mass recovery after a period of immobilization. However, these nutritional interventions should also target muscle functional properties. Yet, muscle mass and force/power production capacities are not always linearly related. For instance, Warren et al. [24] reported a dramatic loss of muscle mass without any change in muscle function after 28 days of hindlimb unloading. Besides, Papadakis et al. [25] reported an increased muscle mass but not change in force production after a pharmacological intervention. Finally, Jacobsen et al. [26] reported an increased force capacity without any change in muscle mass. Muscle mass evaluation is not sufficient on its own, since muscle strength is a better clinical outcome that muscle mass [27]. In practice, a linear relationship between muscle strength and incident disability for activities of daily living has also been reported [28]. Therefore, it seems mandatory to evaluate the effect of any nutritional intervention not only on muscle mass, but also on muscle functional properties, *i.e.* force, power and fatigability. This is particularly relevant in the recovery period after an immobilization, since hypoactivity has adverse effects on muscle maximum force and power production, and fatigue resistance [24], [29]. To our knowledge, the effect of whey proteins supplementation on the recovery of muscle functional properties after an immobilization has never been evaluated, despite this protein source seems to represent a major lever in the rehabilitation of immobilized patients.

## Materials and Methods

### Animals and experimental design – ethics statement

The present study was approved by the Animal Care and Use Committee of Auvergne (CEMEAAuvergne; Permit Number: CE29-11) and adhered to the current legislation on animal experimentation in France. Adult male Wistar rats aged 10−11 months were housed individually under controlled environmental conditions (room temperature 22°C; 12 h light-dark cycle, light period starting at 08:00 h), fed ad libitum a standard 13% casein diet **(** **Table 1** **)** and given free access to water. After a 3-week adaptation period, the functional properties of the plantarflexor muscles were evaluated in vivo under anesthesia. After this testing session, the rats were anesthetized with isoflurane inhalation and subjected to unilateral hindlimb cast immobilization with an Orfit-soft plaque (Gibaud, France) for 8 days (I8). The foot was positioned in plantar flexion to induce a maximal atrophy of the gastrocnemius muscle [7]. For muscle recovery, casts were removed and an evaluation of the in vivo functional properties of the plantarflexors was conducted at 7 (R7), 21 (R21), and 42 (R42) days after cast removal. All rats were fed the standard 13% casein diet during immobilization. During the recovery period, half of the animals were fed the standard 13% casein diet (CAS, n = 9), the other half received a 13% whey protein diet (WHEY, n = 10) **(** **Table 1** **).** As casted rats reduced their food intake during the immobilization period, control non-casted rats were pair-fed (PF, n = 9) to the casted groups at each time point studied and were fed the standard 13% casein diet.

**Table 1:** Composition of the standard and experimental diets.

Ingredients	Standard 13% casein diet	Experimental 13% whey protein diet
Casein 1	166	-
Whey 1	-	144
L-cystine 2	1.8	-
L-proline 2	-	4.7
Colza oil	30	30
Peanut oil	27	27
Sunflower oil	3	3
Cellulose	35	35
Saccharose	100	100
Lactose 3	50	38
Wheat flour	542.2	573.3
Mineral mixture AIN93	35	35
Vitamins mixture AIN93	10	10

1 Casein and whey protein sources are from Lactalis Ingredients, France. Amounts were calculated according to the % of dry matter and nitrogen content.

2 L- cysteine and L-proline was added to the diet to reach and match the AIN93 recommendation for adult rats in both diets. Amounts were calculated according to the amino acid composition of both proteins used.

3 Lactose was added to the diets to match the lactose content and intake for both diets.

Quantities are expressed in g/kg dry matter.

### Evaluation of the muscle functional properties

The functional properties of the plantarflexor muscles were evaluated *in vivo* on an isokinetic dynamometer specially designed for rats (806D, Aurora Scientific, Canada). The testing protocol was similar to that used by Warren et al. [24].

Rats were anesthetized with an intraperitoneal injection of ketamine and acépromazine (1.5 ml/kg, Imalgene 500; 0.250 ml/kg Vétranquil). During the testing procedures, the rat laid supine on a heating plate with the right foot attached to a footplate connected to a dual mode servomotor (305C-LR, Aurora Scientific, Canada). The knee was clamped in place such that the knee angle was 90°, and the ankle axis of rotation coincided with axis of the motor. To avoid any variation in body temperature, the rectal temperature was monitored and computed by a temperature controller (ATC 1000, World Precision Instruments, USA) that adjusted the temperature of the heating plate to maintain the rectal temperature at 37°C. Stimulation of the rat ankle plantarflexors was done percutaneously via Ag/AgCl surface electrodes (StimCom TS0020, Comepa, France). A constant-current electrical stimulator (DS 7A, Digitimer, United Kingdom) was used to deliver square waves (pulse width  =  1 ms). The stimulator was triggered and controlled with automated scripts by an A/D board (Powerlab 8/35, ADInstruments, Australia) and associated software (LabChart 7.3, ADInstruments, Australia).

The protocol consisted in the evaluation of the torque-frequency relationship in isometric condition, the torque-velocity relationship in concentric condition and the assessment of muscle fatigability in isometric condition [24].

To determine the torque-frequency curve, the ankle angle was set at 90° and the plantarflexors stimulated at frequencies varying from 10 to 200 Hz (10, 20, 30, 40, 50, 60, 70, 80, 100, 125, 150, 175, and 200 Hz). These contractions were 200 ms in length with 45 s between contractions, and done in order of increasing stimulation frequency. The reported isometric torque values were calculated as the peak isometric torque minus resting torque. The average isometric torque was also computed from the torque responses to the different stimulation frequencies.

After 3 minutes of rest, the torque-velocity curve was determined from 11 concentric contractions realized at angular velocities of 50, 100, 200, 300, 400, 500, 600, 700, 800, 900, and 1,000°/s, realized in order of decreasing velocity. These contractions were evoked over a 40°-angular amplitude, centered about the 90° ankle angle (*i.e.*, from 70° to 110°). This movement range was chosen because it coincides closely to that of the ankle during the stance phase of voluntary ambulation (*i.e.*, from 72° to 111°; [30]). The contractions were evoked every 45s by stimulation trains delivered at 175 Hz for only the duration necessary to complete the movement (*i.e.*, 40 ms at 1,000°/s to 800 ms at 50°/s); the 175-Hz frequency was used according to Warren et al. [24] recommendation, as the frequency yielding maximal isometric tetanic torque. A power-velocity curve was then computed from this torque-velocity curve. The average concentric power was also computed from the power outputs at the different angular velocities.

Finally, muscle fatigability was assessed with an isometric protocol adapted from the standard Burke fatigue protocol [31]. It consisted in a repetition of 300-ms stimulation trains at a frequency of 40 Hz, repeated every second for 75s. Maximum and minimum isometric torque values were computed to calculate a fatigability index, as follows:

Fatigability index (%)  =  ((minimum torque – maximal torque)/maximal torque) x 100

Data were recorded with an A/D Board (Powerlab 8/35, ADInstruments, Australia), sampled at a frequency of 2000 Hz and analyzed with the Labchart software (LabChart 7.3, ADInstruments, Australia). This software and the Powerlab system were also used to trigger and control the movement of the isokinetic ergometer with automated scripts during concentric contractions.

### Muscle and body mass measurements

Body mass was monitored daily at the same time of day. Muscle mass of the different hindlimb muscles (soleus, gastrocnemius, extensor digitorum longus, tibialis anterior) was measured at R 42. After sacrifice, these muscles were collected, weighted and immediately frozen in liquid nitrogen.

### Statistics

All descriptive statistics presented in the text and figures are means ± SE. Data were screened for normality of distribution and homogeneity of variances using a Shapiro-Wilk normality test and the Barlett’s test, respectively. Each study variable was then compared between the different instances using a two-way (group x frequency for the torque-frequency relationship; group x velocity for the power-velocity curve) ANOVA with repeated measures over time or a one-way (group for the fatigability index) ANOVA with repeated measures over time. Fisher LSD post hoc tests were used when the ANOVA revealed a significant main effect for any factor or interaction. For all statistical analyses, the level of significance was set at P<0.05.

## Results

### Food intake and animal body weight

**Figure 1:**
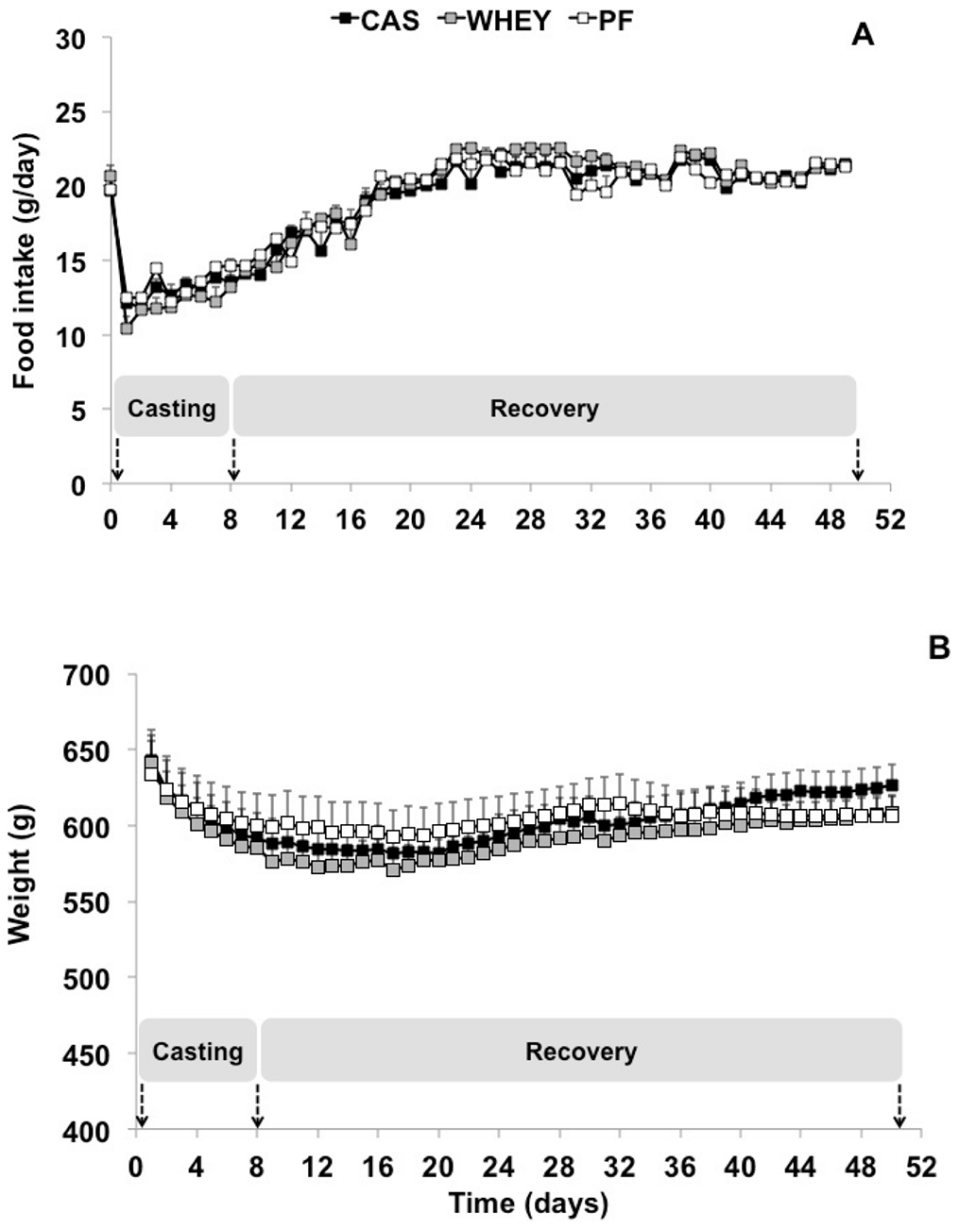
Evolution of food intake and body weight over time: food intake (panel A) and body weight (panel B) of casted rats fed with casein (CAS) or whey (WHEY) and non-casted (PF) rats.

Food intake was similar in all groups before casting and decreased during immobilization to reach 13.6±0.4 and 13.2±1.0 g/d at I8 in the CAS and WHEY groups, respectively (Figure 1). Then, animals increased their food intake during the recovery period. Food intake of the PF group perfectly matched the one of the casted group during immobilization, such that food intake did not differ significantly between the 3 groups during the whole experimental period.

Casted rats exhibited a slight decrease of body weight during immobilization (−9.7±0.5 and −11.6±1.1% at I8 in the CAS and WHEY groups, respectively; P<0.05; Figure 1). Then body weight increased during the recovery period but remained lower than before immobilization at R42 (−3.8±1.1 and −6.7±0.9% in the CAS and WHEY groups, respectively; P<0.05; Figure 1). The PF body weight followed the same modifications with a decrease at I8 and R42 (−8.4±1.1 and −7.4±0.5%, respectively; P<0.05). No significant difference was found in body weights between the 3 groups during the whole experimental period.

### Muscle mass

Plantarflexor muscle masses (soleus + gastrocnemius) were not significantly different between all groups after 42 days of recovery (3.69±0.09, 3.69±0.07 and 3.56±0.07 g for PF, WHEY and CAS, respectively). However, muscle masses varied significantly as a function of group × limb (P = 0.01). In the WHEY and PF groups, the mass of the immobilized plantarflexor muscles was significantly higher at R42 as compared to the non-immobilized limb (WHEY: 3.69±0.07 *vs.* 3.54±0.08 g, P<0.01; PF: 3.69±0.09 *vs.* 3.43±0.07 g, P<0.001). No significant difference was observed between limbs in the CAS group (3.56±0.07 *vs.* 3.52±0.04 g; P = 0.4).

### Torque-frequency relationship

**Figure 2:**
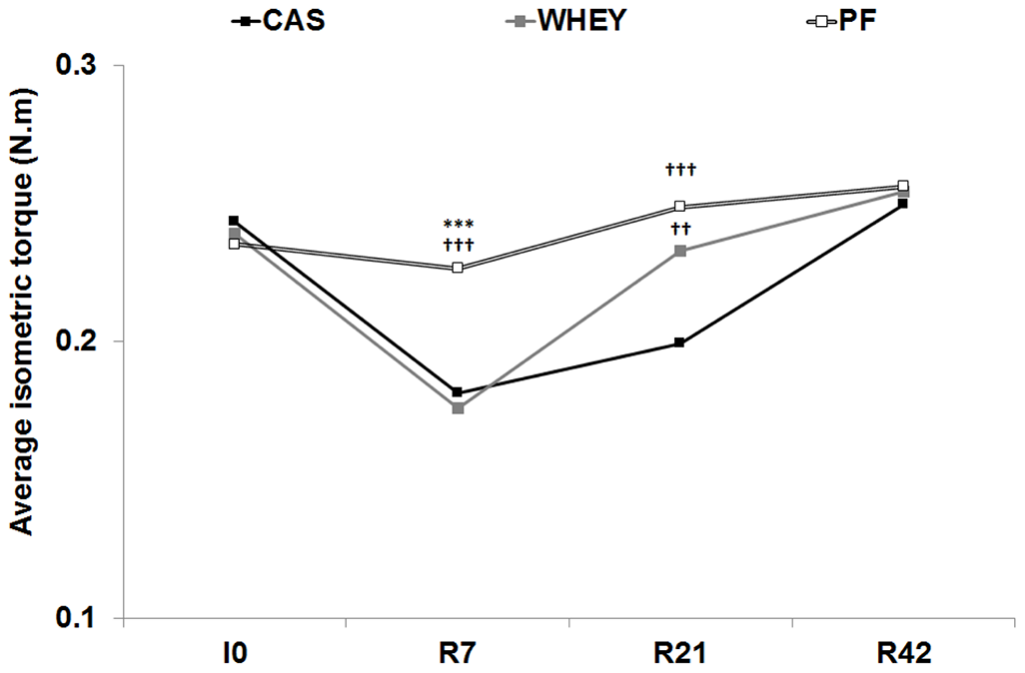
Recovery kinetics of the isometric torque: average isometric torque of the plantarflexors before immobilization (I0) and 7, 21 and 42 days after cast removal (R7, R21 and R42, respectively). Recovery kinetics are shown for the experimental groups fed with casein (CAS), whey (WHEY), and in the pair-fed control group (PF). Significantly different from WHEY: ***: P<0.001. Significantly different from CAS: ††: P<0.01; †††: P<0.001. Standard errors not shown for the sake of clarity.

Average isometric torque decreased similarly in both casted groups at R7 as compared to baseline (CAS: − 19.0±2.6%; WHEY: − 21.7±2.7%; P<0.001; Figure 2), whereas it was not significantly different from I0 in the PF group. Thereafter, torque recovered gradually over time but displayed distinct recovery patterns among the casted groups: at R21, torque was not significantly different from PF in the WHEY group, whereas it remained depreciated in the CAS group (− 11.7±4.0% as compared to baseline; P<0.01). Full recovery was finally observed in the CAS group at R42.

**Figure 3:**
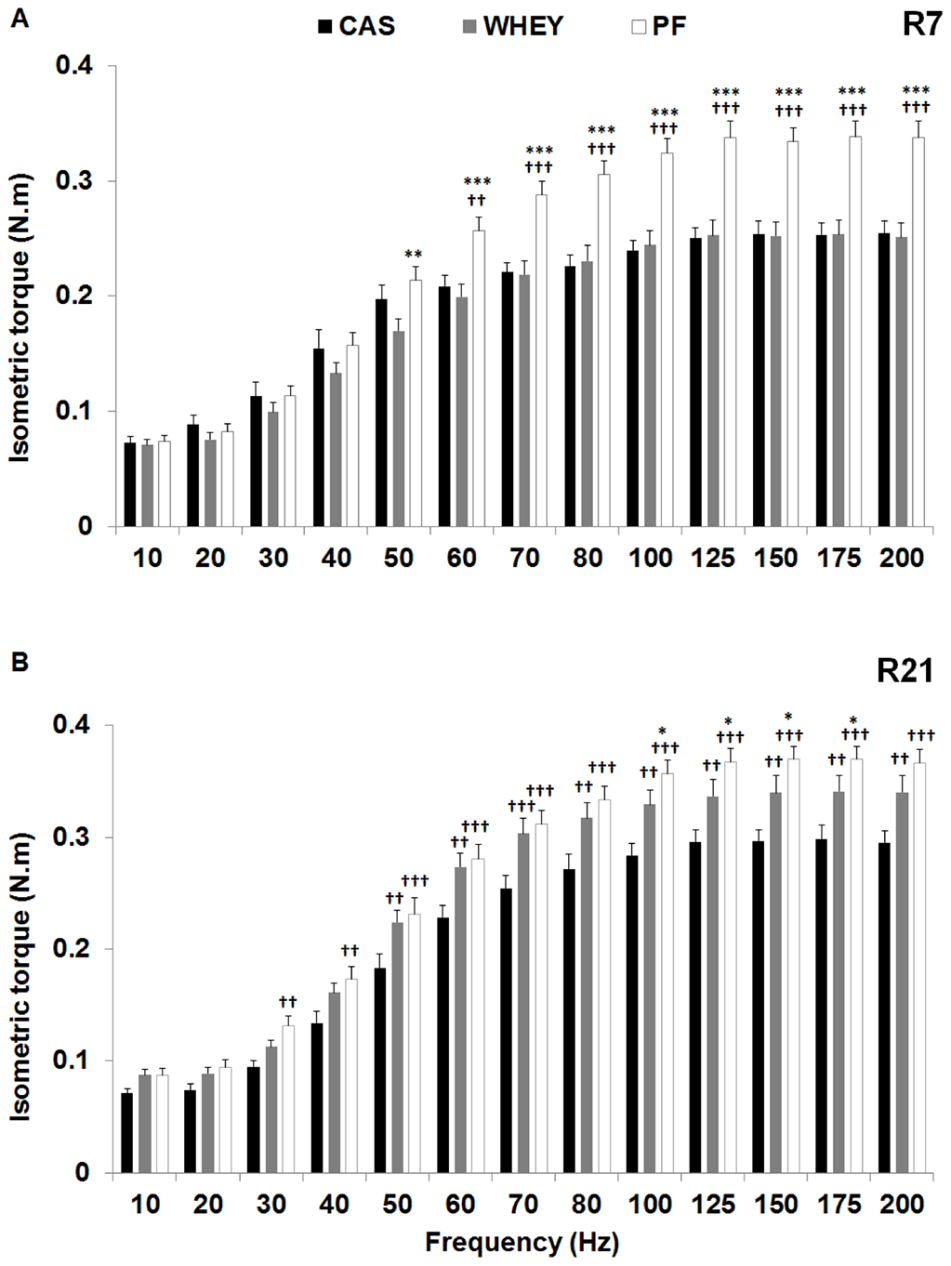
Recovery kinetics of the torque-frequency relationship: torque-frequency relationship after 7 (R7; panel A) and 21 days (R21; panel B) of recovery in the experimental groups fed with casein (CAS; black bars), Whey (WHEY; grey bars), and in the pair-fed control group (PF; white bars). Significantly different from WHEY: **: P<0.01; ***: P<0.001. Significantly different from CAS: ††: P<0.01; †††: P<0.001.

When examining the torque-frequency curve (Figure 3), the same recovery pattern was observed, *i.e.* no difference between casted groups at R7 and a faster recovery at R21 in WHEY as compared to CAS, especially for frequencies above 50 Hz. Nevertheless, torque in the WHEY group remained significantly inferior to PF (P<0.05) in the upper region of the torque-frequency curve, *i.e.* from 100 to 175 Hz. Full recovery was observed in all casted groups at R42.

### Power-velocity relationship

Average power declined after the immobilization period: at R7, power was significantly reduced in both casted groups as compared to baseline (CAS: − 26.8±5.5%, P<0.001; WHEY: − 13.5±6.9%, P<0.05). At R7, average power in the CAS group was also significantly reduced as compared to PF (P<0.05). The same tendency was observed between WHEY and PF (P = 0.07). At R21, data from the casted groups were not significantly different from PF but power was still significantly reduced as compared to baseline in CAS (− 19.6±3.9%, P = 0.001). At R42, average power values in the casted groups were not significantly different from PF. No significant difference was observed between casted groups over time.

**Figure 4:**
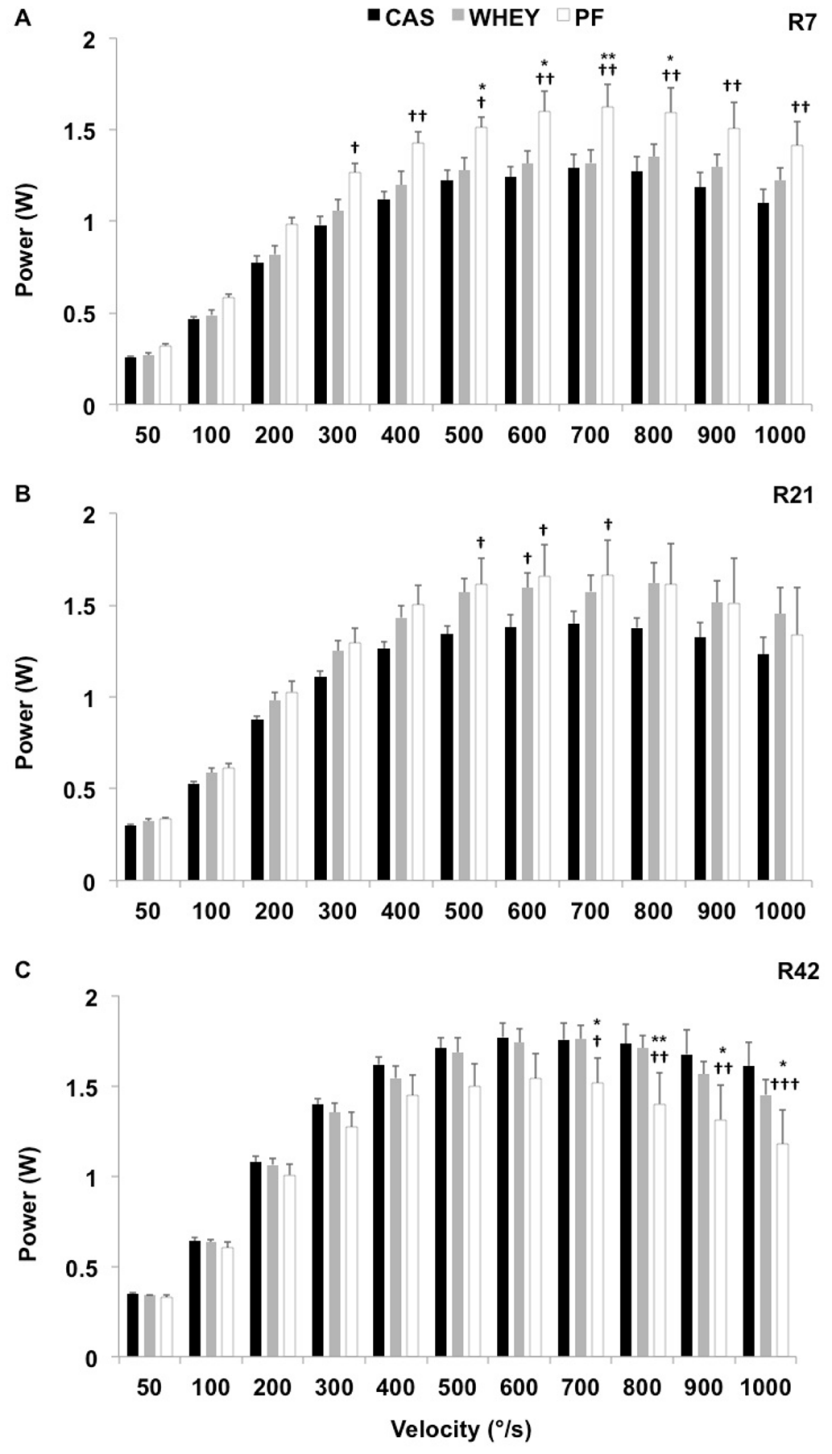
Recovery kinetics of the power-velocity relationship: power-velocity relationship after 7 (R7; panel A), 21 (R21; panel B) and 42 days (R42; panel C) of recovery in the experimental groups fed with casein (CAS; black bars), whey (WHEY; grey bars), and in the pair-fed control group (PF; white bars). Significantly different from WHEY: *: P<0.05; **: P<0.01. Significantly different from CAS: †: P<0.05; ††: P<0.01; †††: P<0.001.

Conversely, some differences between casted groups were observed when examining the complete power-velocity curve (Figure 4). After 21 days of recovery and supplementation (Figure 4B), power values in WHEY were not significantly different from PF, whereas power was still depreciated in CAS for velocities between 500 and 700°/s. Interestingly, peak power was significantly higher in WHEY as compared to CAS (P<0.05). Finally, at R42, power was increased as compared to PF in WHEY and CAS for velocities above 700°/s (Figure 4C). No significant difference was observed between CAS and WHEY at R42.

### Fatigability test

Analysis of variance revealed that the fatigability index was not different among groups: the immobilization procedure and the associated protein supplementation during the recovery period did not affect the fatigability index.

## Discussion

The purpose of this study was to investigate the effect of whey supplementation, as compared to the standard casein diet, on the recovery of muscle functional properties after a casting-induced immobilization period in adult rats. The results revealed that both diets ultimately allowed a complete recovery of skeletal muscle functional properties. However, recovery kinetics varied between diets, the whey diet promoting a 2 times faster recovery of isometric force and concentric power as compared to the casein diet. These effects were more specifically observed at force level and movement velocities that are relevant for functional abilities, and thus natural locomotion.

### Effect of the immobilization procedure

As reported before with this model of immobilization, the food intake and body mass were transiently reduced during the immobilization period and progressively normalized over time (*e.g.* [14]). Conversely, the associated changes in muscle functional properties, *i.e.* force and power, have never been reported after a casting-induced immobilization. Nevertheless, our data can be compared with another model of hypoactivity. Indeed, Warren et al. [24] examined the same parameters after 28 days of hindlimb unloading in rats, and reported significant reductions of isometric torque and concentric power, but no change in fatigability. The highest torque and power deficits were reached 7 days after reloading but recovered within 14 days after reloading. In the current study, we did not find any significant change in muscle fatigability, which is consistent with the findings of Warren et al. [24] and Mc Donald et al. [32]. Although the torque and power deficits felt within the range reported by Warren et al. [24], the time required for full recovery was longer in the present study, *i.e.* 21 to 42 days, but also depended on the quality of the dietary protein supplementation used. In the present study we did not measure functional properties immediately after cast removal, because of the difficulty to set up such measurements at this time point. Indeed, rats generally suffer from oedema and increased stiffness, such that fastening on the isokinetic ergometer may be difficult. Moreover, the first seven days of reloading are generally associated with muscle damage and excitation-contraction uncoupling, which generally worsen the functional deficits [14], [24]. This may have been particularly important in our setting, since rats were immobilized in a shortened position, which favors muscle damage during reloading [33]. However, the torque and power deficits measured at R7 were homogeneous across immobilized groups, such that the differences observed between these groups at R21 can be ascribed to the effect of dietary protein supplementation.

### Effect of protein supplementation

The whey diet clearly promoted a faster recovery of isometric force and concentric power as compared to the casein diet. Such effect could be related to the effect of whey proteins on muscle protein synthesis. Indeed, soluble milk proteins are known to be digested faster than casein, *i.e.* they have a rapid absorption kinetic, but also a higher leucine content [34], [35]. This has been associated with a greater stimulation of protein synthesis at the post prandial state, and thus, on muscle mass gain [7], [36]. Thus, a faster recovery of muscle mass may explain the faster recovery in the group fed with the whey diet. Unfortunately, the current experimental settings do not allow to verify this assumption since muscle mass was only measured at R42. At this time, no difference in plantarflexor muscles mass or in plantar flexion torque was observed between experimental groups. However, there was a clear difference of muscle mass between immobilized and control limbs in the WHEY and PF groups, but not in CAS. The fact that plantarflexor muscles mass was higher in the immobilized limb of the WHEY group is a novel finding with this model of disuse. Previous studies generally reported that muscle mass does not fully recover on the immobilized limb of old animals [12]. In the current experiment, adult rats were studied, which certainly partly accounts for the full recovery of muscle mass. However, the differences observed between the immobilized and non-immobilized muscles masses in the WHEY group also suggests that the muscle contractile activity generated by the electrical stimulation procedures, together with fast proteins supplementation, certainly favored muscle protein synthesis and accretion, as reported previously (*e.g.* [37]). It is however surprising that only 3 sessions of electrical stimulation may have been sufficient to potentiate protein synthesis. However, we cannot rule out the possibility that the benefits of electrical stimulation may have cumulated with an increased spontaneous physical activity, due to the improved muscle functional properties in the WHEY group. The fact that muscle mass did not differ between limbs in the group fed with casein is consistent with other reports showing that these proteins stimulate the muscle protein synthesis to a lesser extent than whey proteins (*e.g.* [37]).

Another important and unexpected result was the increase of concentric power at the higher velocities (> 700°/s) at R42 in the two immobilized groups as compared to the control group. Such phenomenon cannot be ascribed to an increased force, since force did not differ between groups at this point. Therefore, we can only speculate on increased contraction velocity and/or increased muscle-tendon stiffness that may relate with the immobilization and/or reloading processes, since increased power was only observed in the immobilized groups. Increased stiffness has already been reported after a period of immobilization [38] and after contraction-induced injury [39]. This may be linked to the thickening of muscle connective tissue and the up-regulation of collagen synthesis [40], [41]. If such phenomenon occurred, this may have translated into an improved rate of force development, thereby improving power production, especially at the highest velocities, where the rate of force development is crucial, owing to the very short contraction duration.

Alternatively, one may suggest that improved muscle contraction velocity may also have contributed to the increased power production. The first factor that could contribute to an increased contraction velocity is the muscle fiber distribution. However, with the same model of immobilization, Magne et al. [7] did not find any effect of immobilization or diet on the distribution of muscle fiber types. Others reported shifts towards a faster muscle typology, but with longer durations of immobilization, and specifically on muscles with a high proportion of type I muscle fibers, such as the soleus muscle (*e.g.* [42]). However, this muscle has a small contribution to contractile properties in rats, as compared to the gastrocnemius. The fact that the fatigability index was not affected by immobilization certainly rules out the hypothesis of a change in muscle fiber type distribution. Indeed, a shift towards a faster typology may have translated into an enhanced fatigability. The second factor that could contribute to an improved contraction velocity would be altered enzymatic activities within the glycolytic pathway. Interestingly, some studies have reported an improved LDH activity in IIb muscle fibers after a period of immobilization [43], [44]. Additionally, Mercier et al. [45] reported that such effect could be potentiated by the application of electrical stimulation. Unfortunately, we do not have any data to provide evidence for this effect. Thus, additional measurements are required to confirm this hypothesis in our model of disuse. Finally, it is interesting to note that the rats were immobilized at a short muscle length. This may have favored a reduction of the number of sarcomeres in series during the immobilization period (*e.g.* [46]), which may have subsequently translated into enhanced muscle damage after reloading [33]. Interestingly, one of the adaptations to contraction-induced injury is the increase in the number of sarcomeres in series [47]. Such adaptation could have contributed to the increased contraction velocity, and therefore power production. However, additional studies are required to clarify this issue.

### Functional relevance

To reflect as accurately as possible the muscle functional properties, muscle torque was evaluated at different frequencies, and especially those corresponding to the spontaneous firing rates of the rat gastrocnemius muscle, which are typically in the 55–86 Hz range during unforced ambulation [48]. Thus, this frequency range in the torque-frequency relationship is probably the most physiologically relevant one to examine because fast units make up 92% of the gastrocnemius muscle mass and, in turn, the gastrocnemius muscle accounts for 78% of the plantarflexor muscle mass [49]. The faster recovery of torque after whey supplementation at R21 was particularly observed for frequencies above 50 Hz. Therefore, we can reasonably assume that this faster recovery could be particularly relevant from a functional point of view. Similarly, the power produced at velocities between 500 and 700°/s recovered faster in the WHEY group as compared to the group fed with casein. This may also be functionally relevant since the ankle angular velocity measured during voluntary ambulation in rats (averaged over the entire push-off phase, *i.e.*, while the ankle plantarflexors are contracting concentrically), is 300–350°/s, and instantaneous velocity can approach 600°/s [30]. Conversely, the increased power output measured at higher velocities at R42 (>700°/s) may not translate into major benefits for the rat natural locomotion, since such ankle angular velocities may only be required during very quick movements.

## Conclusion

We have shown that the supplementation with whey or casein proteins allowed a complete recovery of functional properties 42 days after a casting-induced immobilization. However, recovery kinetics varied between diets, *i.e.* the whey diet promoting a faster recovery of isometric force and concentric power output as compared to the casein diet. These effects were more specifically observed at force level and movement velocities that are relevant for functional abilities, and thus natural locomotion, in this animal model. Further studies should evaluate the efficacy of whey supplementation in human subjects. If the same conclusions are drawn, the whey diet may be particularly recommended for rehabilitation, in particular in older patients, who are at risk of hypoactivity, and have a reduced ability to recover muscle mass after immobilization periods.

## References

[1] Blottner D, Salanova M, Puttmann B, Schiffl G, Felsenberg D, et al (2006) Human skeletal muscle structure and function preserved by vibration muscle exercise following 55 days of bed rest. Eur J Appl Physiol 97: 261–271. 16568340 10.1007/s00421-006-0160-6

[2] Kortebein P, Ferrando A, Lombeida J, Wolfe R, Evans WJ (2007) Effect of 10 days of bed rest on skeletal muscle in healthy older adults. JAMA 297: 1772–1774. 17456818 10.1001/jama.297.16.1772-b

[3] Pavy-Le Traon A, Heer M, Narici MV, Rittweger J, Vernikos J (2007) From space to Earth: advances in human physiology from 20 years of bed rest studies (1986−2006). Eur J Appl Physiol 101: 143–194. 17661073 10.1007/s00421-007-0474-z

[4] Ferrando AA, Lane HW, Stuart CA, Davis-Street J, Wolfe RR (1996) Prolonged bed rest decreases skeletal muscle and whole body protein synthesis. Am J Physiol 270: E627–633. 8928769 10.1152/ajpendo.1996.270.4.E627

[5] Gibson JN, Halliday D, Morrison WL, Stoward PJ, Hornsby GA, et al (1987) Decrease in human quadriceps muscle protein turnover consequent upon leg immobilization. Clin Sci (Lond) 72: 503–509. 2435445 10.1042/cs0720503

[6] Gibson JN, Smith K, Rennie MJ (1988) Prevention of disuse muscle atrophy by means of electrical stimulation: maintenance of protein synthesis. Lancet 2: 767–770. 2901612 10.1016/s0140-6736(88)92417-8

[7] Magne H, Savary-Auzeloux I, Migne C, Peyron MA, Combaret L, et al (2012) Contrarily to whey and high protein diets, dietary free leucine supplementation cannot reverse the lack of recovery of muscle mass after prolonged immobilization during ageing. J Physiol 590: 2035–2049. 22351629 10.1113/jphysiol.2011.226266 PMC3573319

[8] Paddon-Jones D, Sheffield-Moore M, Cree MG, Hewlings SJ, Aarsland A, et al (2006) Atrophy and impaired muscle protein synthesis during prolonged inactivity and stress. J Clin Endocrinol Metab 91: 4836–4841. 16984982 10.1210/jc.2006-0651

[9] Glover EI, Yasuda N, Tarnopolsky MA, Abadi A, Phillips SM (2010) Little change in markers of protein breakdown and oxidative stress in humans in immobilization-induced skeletal muscle atrophy. Appl Physiol Nutr Metab 35: 125–133. 20383222 10.1139/H09-137

[10] Ikemoto M, Nikawa T, Takeda S, Watanabe C, Kitano T, et al (2001) Space shuttle flight (STS-90) enhances degradation of rat myosin heavy chain in association with activation of ubiquitin-proteasome pathway. FASEB J 15: 1279–1281. 11344113 10.1096/fj.00-0629fje

[11] Jones SW, Hill RJ, Krasney PA, O'Conner B, Peirce N, et al (2004) Disuse atrophy and exercise rehabilitation in humans profoundly affects the expression of genes associated with the regulation of skeletal muscle mass. FASEB J 18: 1025–1027. 15084522 10.1096/fj.03-1228fje

[12] Magne H, Savary-Auzeloux I, Vazeille E, Claustre A, Attaix D, et al (2011) Lack of muscle recovery after immobilization in old rats does not result from a defect in normalization of the ubiquitin-proteasome and the caspase-dependent apoptotic pathways. J Physiol 589: 511–524. 21115641 10.1113/jphysiol.2010.201707 PMC3055540

[13] Taillandier D, Aurousseau E, Meynial-Denis D, Bechet D, Ferrara M, et al (1996) Coordinate activation of lysosomal, Ca 2+-activated and ATP-ubiquitin-dependent proteinases in the unweighted rat soleus muscle. Biochem J 316 ( Pt 1): 65–72. 10.1042/bj3160065 PMC1217351 8645234

[14] Vazeille E, Codran A, Claustre A, Averous J, Listrat A, et al (2008) The ubiquitin-proteasome and the mitochondria-associated apoptotic pathways are sequentially downregulated during recovery after immobilization-induced muscle atrophy. Am J Physiol Endocrinol Metab 295: E1181–1190. 18812460 10.1152/ajpendo.90532.2008

[15] Ferrando AA, Tipton KD, Bamman MM, Wolfe RR (1997) Resistance exercise maintains skeletal muscle protein synthesis during bed rest. Journal of Applied Physiology 82: 807–810. 9074967 10.1152/jappl.1997.82.3.807

[16] Anthony JC, Anthony TG, Kimball SR, Vary TC, Jefferson LS (2000) Orally administered leucine stimulates protein synthesis in skeletal muscle of postabsorptive rats in association with increased eIF4F formation. J Nutr 130: 139–145. 10720160 10.1093/jn/130.2.139

[17] Atherton PJ, Etheridge T, Watt PW, Wilkinson D, Selby A, et al (2010) Muscle full effect after oral protein: time-dependent concordance and discordance between human muscle protein synthesis and mTORC1 signaling. Am J Clin Nutr 92: 1080–1088. 20844073 10.3945/ajcn.2010.29819

[18] Buse MG, Reid SS (1975) Leucine. A possible regulator of protein turnover in muscle. J Clin Invest 56: 1250–1261. 1237498 10.1172/JCI108201 PMC301988

[19] Dardevet D, Sornet C, Balage M, Grizard J (2000) Stimulation of in vitro rat muscle protein synthesis by leucine decreases with age. J Nutr 130: 2630–2635. 11053498 10.1093/jn/130.11.2630

[20] Baptista IL, Leal ML, Artioli GG, Aoki MS, Fiamoncini J, et al (2010) Leucine Attenuates Skeletal Muscle Wasting Via Inhibition of Ubiquitin Ligases. Muscle & Nerve 41: 800–808. 20082419 10.1002/mus.21578

[21] Paddon-Jones D, Sheffield-Moore M, Urban RJ, Sanford AP, Aarsland A, et al (2004) Essential amino acid and carbohydrate supplementation ameliorates muscle protein loss in humans during 28 days bedrest. Journal of Clinical Endocrinology & Metabolism 89: 4351–4358. 15356032 10.1210/jc.2003-032159

[22] Ferrando AA, Paddon-Jones D, Hays NP, Kortebein P, Ronsen O, et al (2010) EAA supplementation to increase nitrogen intake improves muscle function during bed rest in the elderly. Clinical Nutrition 29: 18–23. 19419806 10.1016/j.clnu.2009.03.009

[23] Dardevet D, Remond D, Peyron MA, Papet I, Savary-Auzeloux I, et al (2012) Muscle wasting and resistance of muscle anabolism: the "anabolic threshold concept" for adapted nutritional strategies during sarcopenia. ScientificWorldJournal 2012: 269531. 23326214 10.1100/2012/269531 PMC3541599

[24] Warren GL, Stallone JL, Allen MR, Bloomfield SA (2004) Functional recovery of the plantarflexor muscle group after hindlimb unloading in the rat. Eur J Appl Physiol 93: 130–138. 15248071 10.1007/s00421-004-1185-3

[25] Papadakis MA, Grady D, Black D, Tierney MJ, Gooding GA, et al (1996) Growth hormone replacement in healthy older men improves body composition but not functional ability. Ann Intern Med 124: 708–716. 8633830 10.7326/0003-4819-124-8-199604150-00002

[26] Jacobsen DE, Samson MM, Kezic S, Verhaar HJ (2007) Postmenopausal HRT and tibolone in relation to muscle strength and body composition. Maturitas 58: 7–18. 17576043 10.1016/j.maturitas.2007.04.012

[27] Lauretani F, Russo CR, Bandinelli S, Bartali B, Cavazzini C, et al (2003) Age-associated changes in skeletal muscles and their effect on mobility: an operational diagnosis of sarcopenia. J Appl Physiol 95: 1851–1860. 14555665 10.1152/japplphysiol.00246.2003

[28] Al Snih S, Markides KS, Ottenbacher KJ, Raji MA (2004) Hand grip strength and incident ADL disability in elderly Mexican Americans over a seven-year period. Aging Clin Exp Res 16: 481–486. 15739601 10.1007/BF03327406

[29] Clark BC (2009) In vivo alterations in skeletal muscle form and function after disuse atrophy. Med Sci Sports Exerc 41: 1869–1875. 19727027 10.1249/MSS.0b013e3181a645a6

[30] Varejao AS, Cabrita AM, Meek MF, Bulas-Cruz J, Gabriel RC, et al (2002) Motion of the foot and ankle during the stance phase in rats. Muscle Nerve 26: 630–635. 12402284 10.1002/mus.10242

[31] Burke RE, Levine DN, Tsairis P, Zajac FE 3rd (1973) Physiological types and histochemical profiles in motor units of the cat gastrocnemius. J Physiol 234: 723–748. 4148752 10.1113/jphysiol.1973.sp010369 PMC1350696

[32] McDonald KS, Delp MD, Fitts RH (1992) Fatigability and blood flow in the rat gastrocnemius-plantaris-soleus after hindlimb suspension. J Appl Physiol 73: 1135–1140. 1400027 10.1152/jappl.1992.73.3.1135

[33] Morgan DL (1990) New insights into the behavior of muscle during active lengthening. Biophys J 57: 209–221. 2317547 10.1016/S0006-3495(90)82524-8 PMC1280663

[34] Boirie Y, Dangin M, Gachon P, Vasson MP, Maubois JL, et al (1997) Slow and fast dietary proteins differently modulate postprandial protein accretion. Proc Natl Acad Sci U S A 94: 14930–14935. 9405716 10.1073/pnas.94.26.14930 PMC25140

[35] Dangin M, Boirie Y, Garcia-Rodenas C, Gachon P, Fauquant J, et al (2001) The digestion rate of protein is an independent regulating factor of postprandial protein retention. Am J Physiol Endocrinol Metab 280: E340–348. 11158939 10.1152/ajpendo.2001.280.2.E340

[36] Pennings B, Boirie Y, Senden JM, Gijsen AP, Kuipers H, et al (2011) Whey protein stimulates postprandial muscle protein accretion more effectively than do casein and casein hydrolysate in older men. Am J Clin Nutr 93: 997–1005. 21367943 10.3945/ajcn.110.008102

[37] Tang JE, Moore DR, Kujbida GW, Tarnopolsky MA, Phillips SM (2009) Ingestion of whey hydrolysate, casein, or soy protein isolate: effects on mixed muscle protein synthesis at rest and following resistance exercise in young men. J Appl Physiol 107: 987–992. 19589961 10.1152/japplphysiol.00076.2009

[38] Gillette PD, Fell RD (1996) Passive tension in rat hindlimb during suspension unloading and recovery: muscle/joint contributions. J Appl Physiol 81: 724–730. 8872639 10.1152/jappl.1996.81.2.724

[39] Hoang PD, Herbert RD, Gandevia SC (2007) Effects of eccentric exercise on passive mechanical properties of human gastrocnemius in vivo. Med Sci Sports Exerc 39: 849–857. 17468585 10.1249/MSS.0b013e318033499b

[40] Crameri RM, Langberg H, Teisner B, Magnusson P, Schroder HD, et al (2004) Enhanced procollagen processing in skeletal muscle after a single bout of eccentric loading in humans. Matrix Biol 23: 259–264. 15296940 10.1016/j.matbio.2004.05.009

[41] Slimani L, Micol D, Amat J, Delcros G, Meunier B, et al (2012) The worsening of tibialis anterior muscle atrophy during recovery post-immobilization correlates with enhanced connective tissue area, proteolysis, and apoptosis. Am J Physiol Endocrinol Metab 303: E1335–1347. 23032683 10.1152/ajpendo.00379.2012

[42] Ishihara A, Oishi Y, Roy RR, Edgerton VR (1997) Influence of two weeks of non-weight bearing on rat soleus motoneurons and muscle fibers. Aviat Space Environ Med 68: 421–425. 9143753

[43] Groskreutz JJ, Thompson LV (2002) Enzymatic alterations in single type IIB skeletal muscle fibers with inactivity and exercise in 12- and 30-month-old rats. Aging Clin Exp Res 14: 347–353. 12602568 10.1007/BF03324461

[44] Washington TA, Reecy JM, Thompson RW, Lowe LL, McClung JM, et al (2004) Lactate dehydrogenase expression at the onset of altered loading in rat soleus muscle. J Appl Physiol 97: 1424–1430. 15358753 10.1152/japplphysiol.00222.2004

[45] Mercier C, Lacaille M, Simard C (1993) Influence of electrical stimulation on enzymatic activity of old rat muscles during hindlimb suspension. Mech Ageing Dev 68: 117–124. 8350652 10.1016/0047-6374(93)90144-g

[46] Baker JH, Matsumoto DE (1988) Adaptation of skeletal muscle to immobilization in a shortened position. Muscle Nerve 11: 231–244. 3352658 10.1002/mus.880110308

[47] Lynn R, Talbot JA, Morgan DL (1998) Differences in rat skeletal muscles after incline and decline running. J Appl Physiol 85: 98–104. 9655761 10.1152/jappl.1998.85.1.98

[48] Gorassini M, Eken T, Bennett DJ, Kiehn O, Hultborn H (2000) Activity of hindlimb motor units during locomotion in the conscious rat. J Neurophysiol 83: 2002–2011. 10758110 10.1152/jn.2000.83.4.2002

[49] Armstrong RB, Phelps RO (1984) Muscle fiber type composition of the rat hindlimb. Am J Anat 171: 259–272. 6517030 10.1002/aja.1001710303

